# Prevalence and correlates of hypertension and diabetes mellitus in an urban community in North-Western Nigeria

**DOI:** 10.11604/pamj.2018.29.97.14191

**Published:** 2018-01-31

**Authors:** Beatrice Ohunene Bello-Ovosi, Sunday Asuke, Shehu Ozovehe Abdulrahman, Muhammed Sani Ibrahim, Joseph Ogirima Ovosi, Modupe Arinola Ogunsina, Felicia Ohunene Anumah

**Affiliations:** 1 Department of Internal Medicine, Ahmadu Bello University Teaching Hospital, Zaria, Nigeria; 2 Department of Community Medicine, Bingham University, Jos, Nigeria; 3 Department of Chemical Pathology, Ahmadu Bello University Teaching Hospital, Zaria, Nigeria; 4 Department of Community Medicine, Ahmadu Bello University Teaching Hospital, Zaria; 5 461 Nigerian Air Force Hospital, Kaduna, Nigeria; 6 Department of Internal Medicine, Kaduna State University/BarauDikko Teaching Hospital, Kaduna, Nigeria; 7 Department of Internal Medicine, University of Abuja Teaching Hospital, Gwagwalada, Abuja, Nigeria

**Keywords:** Prevalence, hypertension, diabetes mellitus

## Abstract

**Introduction:**

Worldwide, hypertension and diabetes mellitus (DM) are major causes of morbidity and mortality. This study assesses the prevalence and correlates of hypertension and DM in an urban community in northwestern Nigeria.

**Methods:**

This was a cross-sectional descriptive study. Adults aged 18 years and above, who attended a medical outreach program were interviewed and screened for hypertension and DM. Anthropometry, blood glucose and blood pressure were measured with standard instruments and methodology. Primary outcomes were hypertension and DM. Data were analyzed using STATA version 14 and presented as mean ± standard deviation and frequencies. Chi-square and Pearson's correlation co-efficient were used to identify the correlates of hypertension and DM, at 5% level of significance.

**Results:**

The mean age of participants was 51.0 ± 14.0 years and 87.8% were females. Prevalence of hypertension and DM were 55.9% and 23.3% respectively. Age greater than 40 years and female gender were associated with risk of hypertension and DM respectively, p < 0.05. There was a weak correlation between systolic hypertension and age (r = 0.18, p = 0.02), diastolic hypertension and body mass index (r = 0.16, p = 0.03) and blood sugar and waist circumference (r = 0.19, p = 0.02).

**Conclusion:**

The high prevalence of hypertension and DM among the study population highlights the need for the development and implementation of a community-based public health interventions aimed at reducing their risk factors.

## Introduction

The incidence of non-communicable diseases (NCDs) has been on the rise and Africa bears a disproportionate burden of this increase. Cardiovascular diseases and diabetes mellitus accounts for 48% (18.2 million) and 3.5% (1.33 million) respectively of the 38 million deaths due to NCDs in 2012; sadly, 28 million of these deaths occur in low-and middle-income countries [1, 2]. In Nigeria, one in every five adults die prematurely between the ages of 30 and 70 due to NCDs; and cardiovascular diseases and diabetes mellitus account for 7% and 2% of the 2.08 million deaths attributable to NCDs in 2014 [3, 4]. Hypertension, defined as sustained systemic blood pressure of > 140/90 mmHg, is the single most important modifiable risk factor for cardiovascular morbidity and mortality and is responsible for 18% of global deaths due to NCDs [5]. It affects over 1 billion people worldwide and African regions are disproportionately affected; with a prevalence of 46% in adults aged 25 and above, compared to a prevalence of 35% in Europe and North America [6]. Hypertension is also a major risk factor for stroke and complications of raised blood pressure include: heart failure, peripheral vascular disease, renal impairment and retinal and visual impairment [5, 6]. In Nigeria, the prevalence of hypertension is also on the upward trend. Barely twenty years after the Nigerian National Communicable Disease Survey [7] reported adult prevalence of 11.4% (9.8-14.8%), a systematic review by Akinlua et al [8] reported a prevalence of 17.5-51.6% in urban areas and 4.6-43% in rural areas of Nigeria. Diabetes mellitus (DM) is a chronic, debilitating metabolic disease characterized by chronic hyperglycemia and disturbance of carbohydrate, fat and protein metabolism resulting from defects in insulin secretion and/or insulin action; and associated with a long-term damage and dysfunction of various organs such as the eyes, kidneys, nerves, heart and blood vessels [9]. In 2015, the global prevalence of diabetes mellitus was 8.8%, accounting for 415 million people with the disease; and this was projected to reach 10.4% (642 million with the disease) by 2040, many of which will be from urban settlements [10]. Also, one in two adults with diabetes mellitus are undiagnosed and in Africa, the figure is much higher at 66.7%, therefore, posing a serious risk to the effective management of this disease [10]. Diabetes mellitus has been declared a global emergency of the 21^st^ century because of its rising global prevalence. In Africa, 14.2 million people (prevalence of 3.8%) have diabetes mellitus as at 2015 and this is expected to reach 34.2 million (prevalence of 4.2%) by 2040 [10]. Nigeria is the third most affected country in the region, after Republic of South Africa and Democratic Republic of Congo; and currently has 1.6 million people with diabetes mellitus [10]. The reasons for global explosion in the prevalence of these two diseases, especially in Africa have been adduced to demographic ageing, rapid urbanization and globalization of unhealthy lifestyles that Africa has witnessed in last few decades [6]. Despite this reality, most individuals with hypertension and diabetes are unaware of their state and therefore, are unable to benefit from treatment and preventive measures to avoid complications [10]. Also, at the various governmental levels, the lack of understanding of the enormity of these diseases is still a barrier to effective prevention strategies that could help halt these trends. This study provides data on the burden of hypertension and diabetes mellitus in the urban community of Kawo, Kaduna State in northwest Nigeria.

## Methods

**Study area**: the study was carried out in August 2016 at the District General Hospital, Kawo. Kawo is an urban community located in Kaduna State, Northwest Nigeria. It is made up of four settlement areas: Kawo, Rafinguza, Badarawa and Ungwan Dosa. The inhabitants are mainly civil servants, businessmen, traders and the predominant ethnic group is the Hausa/Fulani [11].

**Study design**: This was a community-based cross-sectional descriptive study.

**Study population**: Subjects were adults of eighteen years and above who are residents of Kawo and who participated in the community outreach program organized by the Nigerian Medical Association, Kaduna State branch.

**Data collection**: Participants who gave informed consent had their socio-demographic data collected via a structured interviewer-administered questionnaire. Physical examination included anthropometric measurements. We measured the weight of each participant to the nearest 0.1kg with a standard weighing scale and height to the nearest 0.5cm with a stadiometer and all were according to standard guidelines and procedures [12]. Body mass index (BMI) was calculated as the ratio of the weight (kg) to the square of the height (meters). Waist circumference was measured at the level of the iliac crest using a flexible non-elastic tape rule and recorded to the nearest 0.1cm [13]. Blood pressure was measured in a sitting position from the non-dominant arm with a mercury sphygmomanometer of appropriate cuff size. Each participant's blood pressure was measured twice, first after 10 minutes of rest and the second, 5 minutes later; and the average was taken. In cases of difference of more than 10mmHg between the first and second measurement, a third measurement was taken 5 minutes later and the average of the three represents the participant's blood pressure. Systolic blood pressure (SBP) and diastolic blood pressure (DBP) were the first and fifth Korotkoff sounds respectively. Random blood glucose was measured using Fine-test meter^®^ (Auto-coding^™^ Premium, Infopia Co. Ltd, South Korea). All procedures were performed under aseptic technique.

**Outcome definitions**: Primary outcomes were hypertension and diabetes mellitus defined as follows: hypertension was defined according to the 7^th^ report of the Joint National Committee on prevention, detection, evaluation and treatment of high blood pressures (JNC VII) thus: < 120/80 mmHg (normal), 120-139/80-89 mmHg (pre-hypertension), 140-159/90-99 mmHg (stage I hypertension) and > 160/100 mmHg (stage II hypertension) or previously diagnosed hypertension on medication [14]. Diabetes was defined according to the American Diabetic Association (ADA) 2016 guidelines as: the presence of osmotic symptoms and random blood sugar above 11.1 mmol/L or previously diagnosed diabetes mellitus on medication [15]. Secondary outcomes were: body mass index classified as overweight (BMI 25.0-29.9 kg/m^2^), obesity (BMI ≥ 30.0 kg/m^2^) [16] and abdominal obesity, defined as waist circumference > 94cm for males and > 80cm for women [13].

**Data analysis**: Data was checked for completeness and validity was ensured by double entry and random checks for errors and outliers. Analysis was done with STATA, version 14 (Stata Corp, College Station, Texas, USA). Numerical variables were expressed as means ± standard deviation, while categorical variables were expressed as frequencies and percentages. 95% Confidence Interval was used to assess the precision of the estimates. Chi-square or Fisher exact test were used to test for relationship between categorical variables. Correlation between blood pressure, blood sugar and anthropometric indices were assessed using Pearson?s co-efficient of correlation. Statistical significance was set at p < 0.05.

**Ethical approval**: Approval for the use of the data was sought and obtained from the Nigerian Medical Association, Kaduna State Branch. To maintain privacy and confidentiality, data was retrieved anonymously without identifiers.

## Results

**Characteristics of the study population**: A total of 181 subjects with age range 23-87 years participated in the study. The mean age of the participants was 51.9 ± 14.0 years. The majority of the participants, 137 (76.5%) were aged > 40 years. One hundred and fifty-nine (87.8%) were females and 127 (70.9%) of the study participants were married. The mean body mass index of the study participants was 21.6 ± 11.3 kg/m^2^while the mean systolic and diastolic blood pressures were 142.4 ± 30.5 and 90.7 ± 16.7mmHg respectively. The mean random blood sugar was 7.0 ± 4.1 mmol/L while the mean waist circumference was 93.5 ± 10.1cm for males and 94.6 ± 14.1cm for the females (Table 1). Overall prevalence of hypertension and diabetes in the community were 55.9% (n = 99; 95% CI 48.3-63.4) and 23.8% (n = 41; 95% CI 18.2-31.4) respectively. The prevalence of hypertension by stage of the disease was 27.4% (n = 49, 95% CI 21.6-34.4) for stage 1 and 28.5% (n = 51, 95% CI 22.5-36.7) for stage 2. Twelve of the participants had both hypertension and diabetes (prevalence of 6.7%; 95% CI 3.5-11.4) (Table 2). The prevalence of hypertension and diabetes mellitus were both associated with age and gender characteristics of the subjects, while no association was found for educational status, ethnicity, employment status, sedentary lifestyle, smoking, alcohol consumption, family history of hypertension and obesity (Table 3, Table 4). There were weak correlations between systolic hypertension and age, r = 0.18, p = 0.02; diastolic hypertension and body mass index, r = 0.16, p = 0.03; and the blood sugar and waist circumference, r = 0.18, p = 0.02 (Table 5).

**Table 1:** Sociodemographic and clinical characteristics of the participants

Characteristics	
*Sociodemographics variables*	*Frequency (%)*
**Age (years)**	
≤ 40	42 (23.5)
> 40	137 (76.5)
**Gender**	
Female	159 (87.8)
Male	22 (12.2)
**Marital status**	
Single	5 (2.8)
Married	127 (70.9)
Widow/widower	43 (24.0)
Divorced/separated	4 (2.2)
**Educational status**	
None	10 (5.5)
Quranic	115 (63.5)
Primary	24 (13.2)
Secondary	20 (11.0)
Tertiary	12 (6.6)
**Ethnicity**	
Hausa/Fulani	149 (86.7)
Others	23 (13.3)
**Employment status**	
Unemployed	104 (61.9)
Employed	64 (38.1)
***Clinical variables***	***Mean ± SD***
Body Mass Index (Kg/m^2^)	26.1± 11.3
**Waist circumference (cm)**	
Female	94.6 ± 14.1
Male	93.5 ± 10.1
**Blood Pressure (mmHg)**	
Systolic	142.4 ± 30.5
Diastolic	90.7 ± 16.7
Random blood sugar (mmol/L)	7.0 ± 4.1

**Table 2:** Prevalence of hypertension and diabetes mellitus among the participants

Variable	Frequency (%)	95% CI
***Blood pressure***		
Normal	31 (17.3)	
Prehypertension	48 (26.8)	
Hypertension	100 (55.9)	48.3-63.4
Stage 1 hypertension	49 (27.4)	21.6-34.4
Stage 2 hypertension	51 (28.5)	22.5-36.7
***Blood Glucose***		
Non-diabetic	131 (76.2)	
Diabetic	41 (23.8)	18.2-31.4
***Combined hypertensive and diabetic***		
No	167 (93.3)	
Yes	12 (6.7)	3.5-11.4

**Table 3:** Prevalence of hypertension by sociodemographic and anthropometric characteristics of the participants

Variable	Hypertensive	Normotensive	P-value
Age group			
≤ 40	17 (9.6)	24 (13.5)	0.046^*^
> 40	81 (45.5)	56 (31.5)	
**Gender**			
Male	6 (3.4)	15 (8.4)	0.009^*^
Female	93 (51.9)	65 (36.3)	
**Educational Status**			
None	7 (3.9)	3 (1.7)	0.153
Quaranic	62 (34.6)	53 (29.6)	
Primary	17 (9.5)	7 (3.9)	
Secondary	10 (5.6)	9 (5.0)	
Tertiary	3 (1.7)	8 (4.5)	
**Ethnicity**			
Hausa/Fulani	82 (48.2)	66 (38.8)	0.468
Others	14 (8.2)	8 (4.7)	
**Employment Status**			
Employed	52 (31.3)	50 (30.1)	0.291
Unemployed	38 (22.9)	26 (15.7)	
**Sedentary lifestyle**			
Yes	30 (17.0)	18 (10.2)	0.244
No	68 (38.4)	61 (34.5)	
**Smoking**			
Yes	1 (0.6)	5 (2.8)	0.053
No	98 (54.8)	75 (41.9)	
**Alcohol**			
Yes	0 (0.0)	2 (1.1)	0.198
No	99 (100)	78 (43.6)	
**Family history of hypertension**			
Yes	45 (25.1)	34 (19.0)	0.692
No	54 (30.2)	46 (25.7)	
**Body Mass Index**			
Normal	18 (11.7)	16 (10.4)	0.235
Overweight	25 (16.2)	27 (17.5)	
Obesity	43 (27.9)	25 (16.2)	
**Central obesity**			
Yes	76 (46.3)	56 (34.2)	0.853
No	19 (11.6)	13 (7.9)	

* Statistical significance, p < 0.05

**Table 4:** Prevalence of diabetes by sociodemographic and anthropometric characteristics of the subjects

Variable	Diabetic	Non-diabetic	P-value
**Age group**			
≤ 40	5 (2.9)	35 (20.6)	0.041^*^
> 40	37 (21.8)	93 (54.7)	
**Gender**			
Male	9 (5.2)	13 (7.6)	0.045^*^
Female	33 (19.2)	117 (68.0)	
**Educational Status**			
None	2 (1.2)	6 (3.5)	0.851
Quaranic	28 (16.3)	81 (47.1)	
Primary	7 (4.1)	17 (9.9)	
Secondary	3 (1.7)	16 (9.3)	
Tertiary	2 (1.2)	10 (5.8)	
**Ethnicity**			
Hausa/Fulani	37 (22.6)	106 (64.6)	0.292
Others	3 (1.8)	18 (10.9)	
**Employment Status**			
Employed	24 (15.0)	75 (46.9)	0.960
Unemployed	15 (9.4)	46 (28.8)	
**Sedentary lifestyle**			
Yes	12 (7.1)	33 (19.5)	0.660
No	29 (17.2)	95 (56.2)	
**Smoking**			
Yes	2 (1.2)	4 (2.3)	
No	40 (23.3)	126 (73.3)	0.454
**Alcohol**			
Yes	0 (0.0)	2 (1.2)	0.198
No	42 (24.4)	128 (74.4)	
**Family history of diabetes**			
Yes	11 (6.4)	20 (11.6)	0.113
No	31 (18.0)	110 (63.9)	
**Body Mass Index**			
Normal	6 (3.9)	29 (19.1)	0.178
Overweight	16 (10.5)	34 (22.4)	
Obesity	13 (8.6)	54 (35.5)	
**Waist circumference**			
Normal	4 (2.6)	27 (17.2)	0.088
Central obesity	33 (21.0)	93 (59.2)	

* Statistical significance, p < 0.05

**Table 5:** Correlation between blood pressure, blood sugar and anthropometric indices

Variable	SBP (r, p-value)	DBP (r, p-value)	Blood sugar (r, p-value)
Age	0.18, 0.02	0.14,0.06	0.10, 0.2
Waist circumference	0.10, 0.2	0.13,0.10	0.18, 0.03
BMI	0.10,0.2	0.16,0.03	-0.10, 0.2
Duration of hypertension	0.14,0.09	0.08,0.33	
Duration of DM			0.12, 0.43

* Statistical significance, p < 0.05

## Discussion

The study reports a high prevalence of hypertension and diabetes mellitus among the participants. The prevalence of 55.9% of hypertension underscore the huge burden of the diseases in the community, which is higher than the reported prevalence in other urban communities in Nigeria [17-19] and higher than the African prevalence of 46% reported by WHO [6]. A meta-analysis by Akinula et al [8] showed a comparable crude prevalence of hypertension in adults of 47.2% (95% CI 43.6-50.8) and 17.5-51.6% in Nigerian urban communities. The prevalence of hypertension by sex was 51.9% in females and 3.4% in males. Studies by Murthy et al [20] and Ezenwaka et al [21] have also shown a slightly higher prevalence of hypertension in the female gender, but not to the extent shown in our study. This higher prevalence in the female population in our study may be partly due to the disproportionately higher number of female participants in the study. The prevalence of 23.3% of diabetes mellitus in our study is also higher than the reported global prevalence of 8.8% and the 2.3% (1.7-5.5) reported for Nigeria [10]. Other studies in Nigeria have also shown prevalence in the range of 3.3-7.7% [22-24]. The relatively higher prevalence in this study may be explained by the fact that the outreach was a screening exercise with initial treatment package before referral. Such situations may have attracted individuals who already knew their hypertensive or diabetic status. The findings of a significant but weak positive correlation between systolic hypertension and age, diastolic hypertension and body mass index and blood sugar with waist circumference in this study have been reported by previous studies [22, 23, 25, 26].

**Limitations of the study**: The diagnosis of hypertension was based on a single contact with the participants and while that of diabetes mellitus was based on random blood sugar. These measurements could have been improved upon if further contacts were made with the patients in the case of hypertension and if fasting blood sugar or glycosylated hemoglobin measurements were also done. Additionally, the outreach included free distribution of anti-hypertensive and anti-diabetic medications before referral and this could have attracted individuals who already had these conditions. Nevertheless, the study provides a somewhat population-based data, which may highlight a better approximation of the situation in the population compared to hospital-based studies.

## Conclusion

In conclusion, there is a high prevalence of hypertension and diabetes in Kawo, an urban community in northwestern Nigeria. This may indicate a rising prevalence of these twin epidemics in other similar communities across the country and therefore, this a call for a more detailed national epidemiological survey of these non-communicable diseases. The findings also highlight the need for public awareness on the risk factors for these diseases and other institutionalized prevention strategies.

### What is known about this topic

- The prevalence of hypertension and diabetes mellitus are rising in Nigeria, especially the urban population;
- In Nigeria, the prevalence of hypertension is slightly higher among the females.

### What this study adds

- The prevalence of hypertension and diabetes mellitus could be higher in Nigeria than previously documented;
- Female gender may be at a higher risk of hypertension than male.

## Competing interests

The authors declare no competing interests.
